# Ramadan Fasting Among Older Children and Adolescents With Type 1 Diabetes Mellitus: A Real-World Study From the UAE

**DOI:** 10.3389/fnut.2022.786678

**Published:** 2022-03-23

**Authors:** Tawfik Muammar, Esphie Grace Fodra Fojas, Radwa Helal, Nader Lessan

**Affiliations:** Imperial College London Diabetes Centre, Abu Dhabi, United Arab Emirates

**Keywords:** Ramadan fasting, type 1 diabetes mellitus, glycaemic control, flash glucose monitoring, continuous glucose monitoring

## Abstract

**Background:**

Ramadan fasting (RF) is a religious obligation for all healthy adult Muslims. The sick and pre-pubertal children are exempt, but many choose to fast for various reasons. In this “real world” study, glycaemic control has been investigated in the context of RF in children and adolescents with type 1 diabetes mellitus (T1DM) and compared multiple daily injections (MDI) and continuous subcutaneous insulin infusion (CSII) outcomes.

**Methods:**

Children and adolescents with T1DM seen at Imperial College London Diabetes Centre who decided to fast in the ensuing Ramadan were educated with their families about diabetes mellitus management during RF using an adapted CHOICE (Carbohydrate, Insulin, and Collaborative Education) educational programme. Pertinent data including hypoglycaemia episodes and diabetic ketoacidosis (DKA) were obtained through patient/family interviews. Information on weight, glycated hemoglobin (HbA1c), and blood glucose levels from continuous glucose monitoring (CGM)/flash glucose monitoring (FGM) before (1 month prior), during, and after (1 month afterwards) Ramadan were retrieved retrospectively from the electronic database. Data are presented as mean ± SD.

**Results:**

Forty-two patients [age 13.5 ± 2.4 years; 27 (64.3%) males; T1DM duration 4.9 ± 3.1 years] were included in the study and were able to fast for 22 ± 9 days during Ramadan. Twenty-three (54.8%) of the patients were on MDI and 19 (45.2%) were on CSII. No statistically significant differences were seen in CGM/FGM generated mean blood glucose level before, during, and after Ramadan [one-way ANOVA (*F*_(2, 80)_ =1.600, *p* = 0.21)]. HbA1c and weight after Ramadan did not change significantly compared to baseline (paired *t*-test; *p* = 0.02 and *p* = 0.08, respectively). Between MDI and CSII groups, there was no significant difference in fasting days (*p* = 0.49), frequency of hypoglycaemia episodes (*p* = 0.98), DKA frequency (*p* = 0.37), HbA1c level (*p* = 0.24), and weight (*p* = 0.11) after Ramadan.

**Conclusion:**

Data show no significant deterioration in indicators of overall glycaemic control which remained inadequate. RF should be discouraged in children with poorly controlled T1DM.

## Introduction

Fasting during the Muslim holy month of Ramadan is one of the five pillars of Islam for all healthy adult Muslims. The fast entails abstinence from eating and drinking from dawn to sunset for a whole lunar month (29 or 30 days). There are no restrictions on food or fluid intake between sunset *(iftar*) and early dawn (*sohour*) (1). The main meal, *iftar*, is taken at sunset and is usually a heavy meal served with deeply fried food and dessert. The other meal, *sohour*, which is taken before sunrise is normally lighter and is primarily composed of complex carbohydrate (2).

Children, the elderly, travelers, pregnant or nursing women, and sick individuals including those with diabetes mellitus, are exempted from fasting. However, many Muslims, both adults and children, including those with diabetes mellitus opt to undertake Ramadan fasting (RF) even when religiously not required, for cultural, social and personal reasons (3). Fasting in people with diabetes mellitus has certain risks including susceptibility to hyperglycaemia after excessive consumption of sweet and fried foods during and after *iftar*, risk of diabetic ketoacidosis (DKA), and hypoglycaemia (4, 5).

Management of type 1 diabetes mellitus (T1DM) among children and adolescents is a challenge, even outside RF periods (6). However, children and adolescents with T1DM are often keen to fast during Ramadan and are able to do so for a significant number of days (6). Amongst users of continuous subcutaneous insulin infusion (CSII) or multiple daily insulin injections (MDI), the main treatment regimens used for older children and adolescents with T1DM, reports indicate no significant difference in glycaemic control with “reasonable risk” of hypoglycaemia and hyperglycaemia (7, 8).

The United Arab Emirates (UAE) has one of the world's highest prevalence rates of diabetes mellitus at 16.3% according to the report of International Diabetes Federation (IDF) in 2019 (7). There are over 1 million people living with diabetes mellitus in the UAE, placing the country in the 15th place worldwide for age-adjusted comparative diabetes mellitus prevalence (9). Studies further point out that the incidence of diabetes mellitus in the UAE is rising at a faster rate than both the MENA (Middle East and Northern Africa) region and the rest of the world (10). Risk factors including rapid economic growth, sedentary lifestyles and unhealthy diets are characteristic to the UAE, and it is expected that there will be 2.2 million individuals with diabetes mellitus in the country by 2040 (7). The incidence of T1DM in the UAE has also risen, making it one of the countries with higher prevalence worldwide (11). In 2019, the new cases were estimated at 20.8 per 1,000 in children and adolescents aged 10–19 years old (12).

Over the last few years, several studies have investigated diabetes mellitus outcomes with RF in adults; studies in the pediatric population in this context have been more limited. This often poses challenges to health care providers in providing medical advice to patients of this age group on the feasibility of RF partly because little is known about the safety or metabolic effects of fasting in this group of patients (13).

The current cohort study investigates the outcomes of RF using MDI and CSII to assess patterns of glycaemic control and severity of complications during RF amongst older children and adolescents with T1DM seen at the Imperial College London Diabetes mellitus Center (ICLDC), UAE. The effects of dose adjustment, health professional team and parental support on safety and glycaemic outcomes in children and adolescents with T1DM during Ramadan were also investigated.

## Patients and Methods

### Study Design and Participants

This was a retrospective study on children and adolescents with T1DM and their families seen at ICLDC who were routinely evaluated in the year 2019. Ramadan began on May 6th, 2019 and ended on June 4th, 2019; RF lasted for 30 days in the UAE with around 14 hours of fasting per day. Study population age range was between 9–18 (13.5 ± 2.4) years and as such included older children and adolescents as defined by the World Health Organization (14).

Approximately one month before Ramadan, participants who resolutely intended to fast during Ramadan were educated about diabetes mellitus management during fasting. Medical team included physicians, nurses, diabetes mellitus educators, dieticians, and telemedicine staff. The CHOICE (Carbohydrate, Insulin, and Collaborative Education) educational programme has been adapted by the pediatric diabetes mellitus and endocrinology clinic in ICLDC and was used to guide before Ramadan education during this study. The programme recommends insulin dose adjustments according to diet and lifestyle changes in Ramadan (15, 16).

The programme focuses firstly on insulin dose adjustments. For insulin pump users who normally have a follow up visit and before Ramadan preparation, patients have their pumps adjusted to a new setting in which insulin dose is decreased during daytime, increased after *iftar*, and decreased before *sohour* to avoid hypoglycaemia. Patients on MDI are instructed to decrease their long-acting insulin by 20%, decrease *sohour* meal short acting insulin by 10–20%, and increase short acting insulin for *iftar* meal by 0–10% according to their needs.

The second arm of the educational programme before Ramadan is dietetic advice. Diet plans are individually designed by dietitians to target consumption of complex carbohydrate rich food in *iftar* meal, with strict recommendations to avoid high sugar containing food and snacks. Patients are further instructed to start their plan of decreasing the long-acting insulin 1 day before the start of Ramadan. Parents' proactive role is essential in closely tracking the blood glucose level of their children in the first couple of days of RF especially after 4 pm (around 2 h before *iftar* meal).

**Figure 1:**
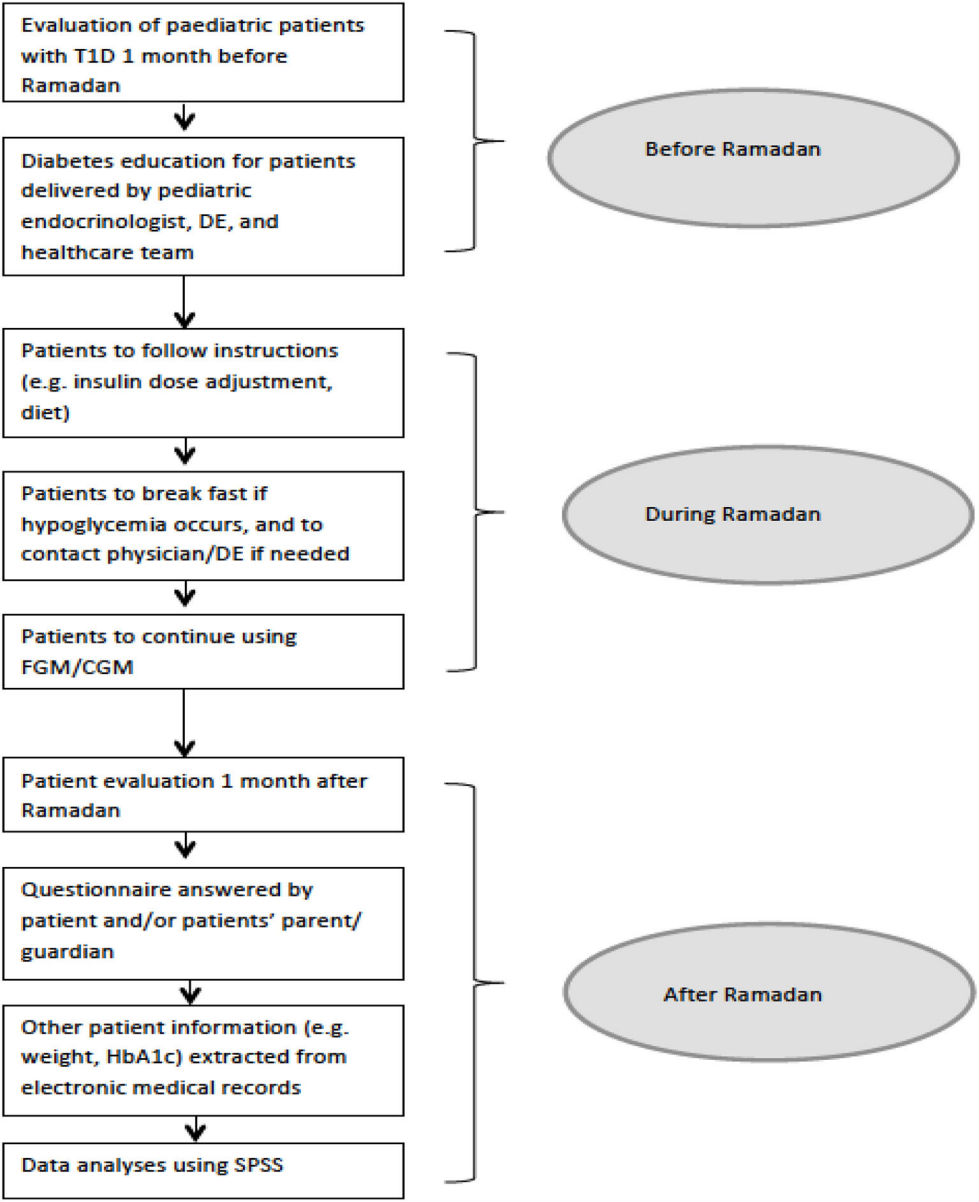
Study flowchart: an overview of study design. T1DM, type 1 diabetes mellitus; DE, diabetes mellitus educator; CGM, continuous glucose monitoring; FGM, flash glucose monitoring; SPSS, Statistical Package for the Social Sciences.

Patients and their parents are strictly advised to break the fast if hypoglycaemia occurs, even if it is only a few minutes before *iftar*, to avoid complications. If the first day passed safely and easily, patients are allowed to continue fasting provided they will follow the recommendations. If the first day had episodes of hypoglycaemia, further reduction of long-acting insulin is recommended with close monitoring, and the physician and/or diabetes mellitus educator can be contacted if needed. The flow chart of the methods is outlined in Figure 1.

### Outcome Measures

The mean fasting hours was 14 h and 40 min as it was at the summer season in UAE. Average temperature was 31°C, while humidity ranged between 45 and 55%.

Approximately one month after RF, a questionnaire was given to the participants (and their families) on their clinic visit after Ramadan. The questionnaire was in English (Arabic translation included) with eight items; questions included age, type of treatment, continuous glucose monitoring/flash glucose monitoring (CGM/FGM) sensors used, days fasted, hypoglycaemia episodes, frequency of DKA, best support noted during the fast, and willingness to fast in the next Ramadan 2020. Information from the centre's database was retrieved for weight, insulin dose, glycated hemoglobin (HbA1c), and blood glucose levels based on uploaded sensor readings. Blood samples collection and weight assessment were usually done during clinic attendance between 7 am-2 pm after 6–8 hours fasting. Patients were grouped and compared according to insulin regimen (MDI vs. CSII) and outcome variables (fasting days, HbA1c and blood glucose levels, weight, hypoglycaemia episodes, insulin dose, and DKA) were analyzed. Written general consent including use of data for research use was provided by the participants and/or participants' parents/legal guardian upon patient registration in the centre.

### Statistical Analysis

Frequency (percent) and mean ± standard deviation (SD) was computed for the numeric and categorical variables. Student's *t*-test and one-way analysis of variance (ANOVA) were used to compare the outcome variables as appropriate. For evaluation of “reasonable” RF risk on glycaemic control, statistical significance was reported for a *p*-value < 0.01. Data analyses were performed using Statistical Package for Social Sciences (SPSS) version 24.

## Results

Forty-seven patients signified intention to fast during Ramadan; data of five (11%) patients were excluded in the analyses because of fasting plans withdrawal. Forty-two (89%) patients were included in the study; fasting days of 22 ± 9 days during Ramadan. All patients' pubertal stage was within normal range for age based on routine clinical assessment. Baseline characteristics of the study cohort are summarized in Table 1. Majority (46.8%) of the participants used flash glucose monitoring FGM flash glucose monitoring (Freestyle Libre) as their glucose monitoring system (36.2% Enlite, 14.9% Accu-chek).

**Table 1:** Baseline characteristics of the study cohort.

**Parameters**	**Total (*n* = 42)**
MDI	23 (54.8%)
CSII	19 (45.2%)
Sex (male)	27 (64.3%)
Age (years)	13.5 ± 2.4
Duration of T1DM (years)	4.9 ± 3.1
Insulin, basal (units/day; *n* = 14)	23.9 ± 10.4
Insulin, bolus (units/meal; *n* = 20)	13.3 ± 7.8

*MDI, multiple daily injections; CSII, continuous subcutaneous insulin infusion; T1DM, type 1 diabetes mellitus*.

**Figure 2:**
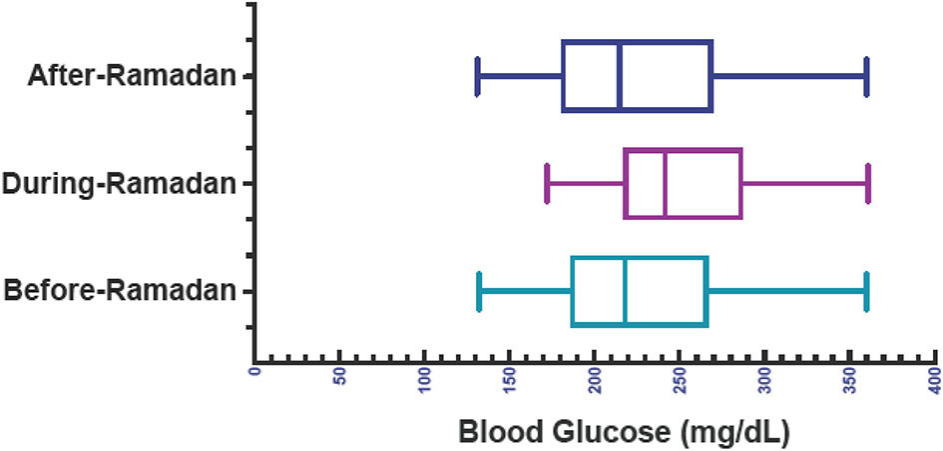
Blood glucose levels before-, during-, and after-Ramadan.

For all participants (*n* = 42), there was no statistically significant difference in mean blood glucose levels before- (226.5 ± 54.6 mg/dL), during- (226.5 ± 54.6 mg/dL), and after- (225.7 ± 58.8 mg/dL) Ramadan [one-way ANOVA; *F*_(2, 80)_ = 1.600, *p* = 0.208; Figure 2]. HbA1c levels (9.0 ± 1.7 vs. 8.7 ± 1.3) and weight (53.3 ± 18.0 vs. 54.9 ± 19.3) before and after Ramadan did not change significantly compared to baseline (paired *t*-test; *p* = 0.02 and *p* = 0.08, respectively).

In the MDI group, there was no significant difference in paired *p*-values for before- and during-Ramadan (*p* = 0.132), before- and after-Ramadan (*p* = 0.865), and during- and after-Ramadan blood glucose levels; similarly, no statistically significant difference was found for the CSII group (*p* = 0.957, *p* = 0.964, *p* = 0.225, respectively). Between the MDI and CSII groups, there was no significant difference in unpaired *p*-values for before- and during-Ramadan (*p* = 0.368), before- and after-Ramadan (*p* = 0.872), and during- and after-Ramadan (*p* = 0.434) (Table 2).

**Table 2:** Changes and comparisons in blood glucose levels before-, during-, and after-Ramadan.

	**Average blood glucose level (mg/dL) before 4-week Ramadan fasting**		**Average blood glucose level (mg/dL) during Ramadan compared with level before 4-week Ramadan fasting**				**Average blood glucose level (mg/dL) after Ramadan compared with level before 4-week Ramadan fasting**				**Average blood glucose level (mg/dL) during Ramadan compared with level after 4-week Ramadan fasting**			
**Cohort**	**Mean**	**SD**	**Mean paired difference^**a**^**	**SD**	**Paired *p*-value^**b**^**	**Unpaired *p*-value^**c**^**	**Mean paired difference^**a**^**	**SD**	**Paired *p*-value^**b**^**	**Unpaired *p*-value^**c**^**	**Mean paired difference^**a**^**	**SD**	**Paired *p*-value^**b**^**	**Unpaired *p*-value^**c**^**
MDI *n* = 23, girls/boys = 8/15, mean (SD) age = 13.1 years (2.6)	222.5	57.9	24.4	40.4	0.132 (*n* = 8)	0.368	−2.3	50.1	0.865 (*n* = 14)	0.872	−9.9	52.9	0.64 (*n* = 7)	0.434
CSII *n* = 19, girls/boys = 7/12, mean (SD) age = 14.1 years (2.1)	230.6	52.7	−1.3	66.1	0.957 (*n* = 8)		0.5	40	0.964 (*n* = 13)		−36.5	56.9	0.225 (*n* = 5)	

a *Paired difference indicates change in blood glucose level for each subject (i.e., positive and negative values indicate increase and decrease in blood glucose level, respectively)*;

b *Paired p-value compares the mean paired differences within cohorts*;

c *Unpaired p-value compares the mean paired differences between the two cohorts. CSII, continuous subcutaneous insulin infusion; MDI, multiple daily injections*.

In addition, between the two groups, there was no significant difference in fasting days (*p* = 0.49), HbA1c levels (*p* = 0.24), and weight (*p* = 0.11) after Ramadan; as well as reported frequencies of hypoglycaemia episodes (*p* = 0.98) and DKA (*p* = 0.37) during Ramadan (Table 3). More than half (57%) of the patients and their families noted dose adjustment as the main support during the fast, followed by parents' support (45%). All (100%) of the patients who completed the fast were willing to fast in the next Ramadan.

**Table 3:** After Ramadan values on fasting days, hypoglycaemia and DKA frequency, HbA1c, and weight: MDI and CSII compared.

**After Ramadan**	**Total (*n* = 42)**	**MDI (*n* = 23)**	**CSII (*n* = 19)**	***P*-value**
Fasting days	22.3 ± 8.7	23.2 ± 8.1	21.3 ± 9.5	0.49
Hypoglycaemia frequency^*^	1.1 ± 2.3	1.1 ± 2.2	1.1 ± 2.5	0.98
DKA frequency^*^	0.02 ± 0.15	0.04 ± 0.21	0 ± 0.00	0.37
HbA1c (%)	8.7 ± 1.3	8.9 ± 1.4	8.4 ± 1.1	0.24
Weight (kg)	54.9 ± 19.3	50.5 ± 20.4	60.1 ± 16.9	0.11

*DKA, diabetic ketoacidosis; HbA1c, glycated hemoglobin; MDI, multiple daily injections; CSII, continuous subcutaneous insulin infusion*;

* *reported for during Ramadan. Data is presented as mean ± SD*.

## Discussion

Previous studies have shown that effective counseling before RF delivered by health care providers reduces the incidence of hypoglycaemia (17). Patient education before Ramadan provides a platform to remind patients about the importance of diet, exercise, dose adjustment and that regular glucose monitoring is vital to avoid complications while reassuring them that this does not invalidate the fast (9). Patient education is certainly a cornerstone of Ramadan diabetes mellitus management (18), and is stringently applied in the ICLDC pediatric clinic. Patients are instructed that the first day of Ramadan is the most important day to carefully assess glycaemic control as it gives a provisional prediction of glucose excursions in the upcoming month of fasting. Other topics discussed during before Ramadan education sessions include prior fasting experiences, ways to reduce blood glucose levels fluctuations and complications, how to improve self-reliance and confidence, tailored change plan for dose and pump programming adjustments, and more importantly, informed decision-making about fasting. This education plan offers an imperative opportunity to allow those who wish to fast to do so more safely (9).

The recent 2021 recommendations of the (IDF)/Diabetes and Ramadan (DAR) Alliance (19) discourage children and adolescents with T1DM to practice RF. However, many children and adolescents choose to fast (20), presenting a challenge to health care providers. Children and adolescents can be even more eager to fast compared to their adult family members, a phenomenon related to self-esteem and happiness (21, 22). The Islamic norm on RF in this age group is for Muslim children to fast when specific features of puberty are attained (21, 23). The challenge to physicians is primarily in providing intensive pre-fasting education and emphasizing the need for closer blood glucose monitoring during the fast (2).

Although several studies have investigated different aspects of RF in individuals with diabetes mellitus, most exclude children and adolescents. The issues and challenges in this age group are, in fact, often more pronounced and, in some ways, different. During childhood, the responsibility on managing diabetes mellitus falls on the parents who usually decide on whether to proceed with the full RF for their children. The parents try to monitor blood glucose fluctuations and make adjustments of insulin doses with primary aim of avoiding hypoglycaemia. As a result, exaggerated hyperglycaemic response may happen and is more pronounced at the time when fasting is broken (*iftar*). In adolescents, the problems can be even more challenging because at this age, they prefer to take charge of their disease management and are often less predictable with their dietary and medication compliance (20, 24).

International guidelines on diabetes mellitus management for adults during RF confirm that poor control of diabetes mellitus is considered as one of the main contraindications for fasting (17, 25–27). Well-controlled children and adolescents with T1DM are expected to have less complications making RF feasible for them (28). Studies on the effects of RF in children with diabetes mellitus have been few and results have been inconsistent. While some studies showed stable HbA1c before and after Ramadan (22, 29), some other studies showed deterioration of glycaemic control in the form of elevated HbA1c after Ramadan (24, 30). Bin-Abbas et al., suggested that fasting can be safe for well-controlled adolescents with T1DM who are using CGM devices (29), whereas other investigators have asserted that RF might predispose to acute complication in patients of this age group (30, 31). Further diabetes mellitus management challenges exist for poorly controlled children and adolescents with T1DM who fast against medical advice, as in some patients in the cohort. Study showed no significant differences were observed on mean blood glucose levels, HbA1c, and weight before- and after-Ramadan. These results need to be interpreted with caution. Our previous studies in adults indicate that HbA1c is a poor indicator of changes in glycaemic control when comparing a one-month time window such as Ramadan with another. A similar principle may be applicable in the pediatric population and that a CGM/FGM-derived glucose management indicator (GMI, or estimated HbA1c) may be better suited for looking at any such potential change more closely. Furthermore, adult studies show definite differences in glucose profiles of individuals with diabetes mellitus during Ramadan when compared with non-Ramadan periods, with a significant glucose excursion at *iftar* time which is more pronounced in people with poorer glycaemic control (32).

The results of this study also support previous studies which show that outcomes of CGM/FGM use with both MDI and CSII during RF are not significantly different. A recent similar study in Kuwait (33) is also in agreement with the present study that intensive monitoring along with patients' education before Ramadan are crucial for safe RF in the cohort. Another study conducted in Saudi Arabia reported that children and adolescents with T1DM using FGM could fast Ramadan without severe hypoglycaemia or DKA risks (34).

### Limitations

This was a single centre study conducted in a specialized diabetes mellitus management centre, and therefore some selection bias may have influenced the glycaemic profile excursion. Moreover, the small sample size of the study might skew the data, as it was done on older children and adolescents with T1DM who decided to fast Ramadan. Importantly, studies established with more CGM data had shed more light on RF-induced effect on glycaemic profiles in adults; such research may also give more depth and breadth in studies on children and adolescents.

## Conclusion

This study sheds light on the practice of RF in children and adolescents which is often a personal decision supported by family, social and cultural values which may not necessarily be supported by religious recommendations. The challenge to healthcare professionals is significant; and this study shows the impact of a modified CHOICE before Ramadan education programme (16) guided by the IDF-DAR recommendations (19) in addition to the expertise of a diverse health care team. More evidence from future studies is needed to improve medical guidelines and help health care providers take proper clinical decisions.

## Data Availability Statement

The raw data supporting the conclusions of this article will be made available by the authors, without undue reservation.

## Ethics Statement

Ethical review and approval was not required for the study on human participants in accordance with the local legislation and institutional requirements. Written general consent including use of data for research use was provided by the participants and/or participants' parents/legal guardian upon patient registration in the centre.

## Author Contributions

TM: study concept, data acquisition, and manuscript editing. EF: statistical analyses and manuscript writing. RH: data interpretation and manuscript writing. NL: study design, data interpretation, and manuscript editing. All authors contributed to the article and approved the submitted version.

## Conflict of Interest

The authors declare that the research was conducted in the absence of any commercial or financial relationships that could be construed as a potential conflict of interest.

## Publisher's Note

All claims expressed in this article are solely those of the authors and do not necessarily represent those of their affiliated organizations, or those of the publisher, the editors and the reviewers. Any product that may be evaluated in this article, or claim that may be made by its manufacturer, is not guaranteed or endorsed by the publisher.
